# Improving Efficiency of Multicrystalline Silicon and CIGS Solar Cells by Incorporating Metal Nanoparticles

**DOI:** 10.3390/ma8105337

**Published:** 2015-10-08

**Authors:** Ming-Jer Jeng, Zih-Yang Chen, Yu-Ling Xiao, Liann-Be Chang, Jianping Ao, Yun Sun, Ewa Popko, Witold Jacak, Lee Chow

**Affiliations:** 1 Department of Electronic Engineering, Chang Gung University, Kweishan-Taoyuan 333, Taiwan; b0027111@stmail.cgu.edu.tw (Z.-Y.C.); m0324016@stmail.cgu.edu.tw (Y.-L.X.); liann@mail.cgu.edu.tw (L.-B.C.); 2 Institute of Photoelectronic Thin Film Devices and Technology and Tianjin Key Laboratory of Thin film Devices and Technology, Nankai University, Tianjin 300071, China; aojp@nankai.edu.cn (J.A.); suny@nankai.edu.cn (Y.S.); 3 Institute of Physics, Wroclaw University of Technology, Wroclaw 50-370, Poland; Ewa.Popko@pwr.edu.pl (E.P.); witold.aleksander.jacak@pwr.edu.pl (W.J.); 4 Department of Physics, University of Central Florida, Orlando, FL 32816, USA; Lee.Chow@ucf.edu

**Keywords:** Au and Ag nanoparticles, multicrystalline silicon, CIGS solar cells, spin coating

## Abstract

This work studies the use of gold (Au) and silver (Ag) nanoparticles in multicrystalline silicon (mc-Si) and copper-indium-gallium-diselenide (CIGS) solar cells. Au and Ag nanoparticles are deposited by spin-coating method, which is a simple and low cost process. The random distribution of nanoparticles by spin coating broadens the resonance wavelength of the transmittance. This broadening favors solar cell applications. Metal shadowing competes with light scattering in a manner that varies with nanoparticle concentration. Experimental results reveal that the mc-Si solar cells that incorporate Au nanoparticles outperform those with Ag nanoparticles. The incorporation of suitable concentration of Au and Ag nanoparticles into mc-Si solar cells increases their efficiency enhancement by 5.6% and 4.8%, respectively. Incorporating Au and Ag nanoparticles into CIGS solar cells improve their efficiency enhancement by 1.2% and 1.4%, respectively. The enhancement of the photocurrent in mc-Si solar cells is lower than that in CIGS solar cells, owing to their different light scattering behaviors and material absorption coefficients.

## 1. Introduction

A surface texture that causes light scattering into a conventional solar cell is an effective means of trapping light in the semiconductor. However, the associated increase in the surface area increases the rate of minority carrier recombination on the surface regions. Recently, the use of metal nanoparticle plasmonics to improve the efficiency of solar cells has been extensively studied [1,2,3,4,5,6,7,8,9,10,11,12,13,14,15,16]. The three well-known methods for increasing photocurrent in solar cells by the incorporation of metal nanoparticles are as follows. First, metal nanoparticles can scatter incident light over a range of angles, increasing the path length of the light in the absorbing semiconductor. Second, the surface plasmonic effect redirects light by the formation of waveguide mode, which is then absorbed in the semiconductor. Third, the localized surface plasmonic resonance of the metal nanoparticle causes high near-field intensities, which enhanced the absorption [12,13,14,15,16]. Properly selecting the size of the metallic nanoparticles and dielectric structures on a semiconductor causes light to be concentrated and folded into a semiconductor because light in a semiconductor scatters preferentially into layers with a high dielectric constant, increasing the absorption of the semiconductor. The three general modes of application of nanoparticles in semiconductor are (i) metal nanoparticles on the surface; (ii) metal nanoparticles in the active layer and (iii) a nano-grating metal on the back contact. The use of metal nanoparticles on the surface or a nano-grating metal on the back contact is more suitable for crystalline silicon solar cells or GaAs solar cells than the other type of solar cells. The embedding of metal nanoparticle in active layer is the most effective means of improving light absorption in dye-sensitized or organic solar cells. Metal nanoparticles on the surface of the semiconductor are effective in thin film solar cells. Photocurrent enhancement by the plasmonic effect in solar cells has been reported for many semiconductors. Zhang *et al.* proposed the use of aluminum (Al) nanoparticles to perform broadband light trapping, improving photon absorption in Si wafers by 28.7% [1]. Derkacs *et al.* demonstrated an improved transmission of electromagnetic radiation as a result of forward scattering by surface plasmon polariton modes in gold (Au) nanoparticles that were deposited above the amorphous silicon solar cells. They achieved an 8.1% improvement in short-circuit current density and an 8.3% improvement in energy conversion efficiency [2]. Liu *et al.* systematically studied surface plasmon resonance on gallium arsenide (GaAs) thin film solar cells on whose surface were variously sized silver (Ag) nanoparticles. They found that the short circuit current density of a GaAs solar cell with Ag nanoparticles was 14.2% higher than that of the untreated solar cells [3]. Nahm *et al.* incorporated Au nanoparticles with diameter of approximately 100 nm into titanium dioxide (TiO_2_) thin films for use in dye-sensitized solar cells (DSSCs), and improved their power-conversion efficiency from 2.7% to 3.3% [6]. Chen *et al.* demonstrated that Au plasmonic nanoparticles at the pn-junction interface of copper-indium-gallium-diselenide (CIGS) /cadmium sulfide (CdS) improved the efficiency of CIGS solar cells from 8.31% to 10.36% [8].

The simplest way to enhance the solar cell efficiency is to incorporate metal nanoparticles on their surface. This method does not alter the process of fabrication of solar cells and does not form recombination center. The design parameters of metal nanoparticles, including nanoparticle size and shape, dielectric environment and the stack layer of semiconductors, strongly influence solar cell performance. Near-field confinement, far-field scattering and light trapping effects have the potential to improve absorption in solar cells, but parasitic absorption loss in metal nanoparticles remains a critical issue. Unfortunately, no simple rule can predict the dominant effect, which can be identified only by solving complicated Maxwell equation or experimentally. To the best of the authors’ knowledge, plasmonic effects have been seldom exploited in CIGS solar cells. Therefore, this study experimentally investigates the use of metal nanoparticle on CIGS solar cells. Multicrystalline silicon solar cells are also examined for comparison. Ag and Au nanoparticles are the most widely used materials because their surface plasmon resonances are located in the visible range and so interact more strongly with the peak solar intensity. The literature includes many methods for incorporating metal nanoparticles, such as e-beam lithography [4], thermal evaporation and annealing [17], chemical bath deposition [18,19], spin coating [20] and nanoimprinting [21], among others. In this work, Au and Ag nanoparticle are simply incorporated into the surface of solar cells.

## 2. Results, Discussion

**Figure 1:**
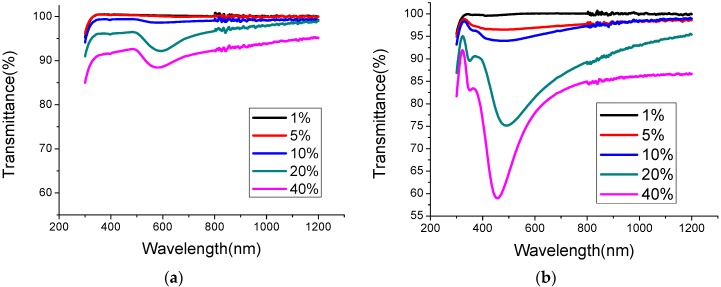
The transmittance of (**a**) Au and (**b**) Ag nanoparticles at concentrations of 1%, 5%, 10%, 20% and 40% on glass surface.

Figure 1a,b present the transmittance of Au and Ag nanoparticles at concentrations of 1%, 5%, 10%, 20% and 40% on glass surface, respectively. The dip in the transmittance yields the plasmonic resonance wavelength and the resonance curves tend to become broader as nanoparticle concentration is reduced. The background transmittance increases as nanoparticle concentration decreases because shadowing effect becomes weaker. The transmittance of Ag nanoparticles is less than that of Au nanoparticles. The resonance wavelengths of Au and Ag nanoparticles at a concentration of 40% are 581 nm and 457 nm, respectively. The broadening of the resonance wavelength is caused by both the random distribution of nanoparticles and the non-uniform particle of their sizes. This broadening behavior is expected to be favorable for solar cell applications, because it causes light-trapping over a wider range of wavelengths. Larger metal nanoparticles result in stronger scattering effect but also greater metal absorption loss. Different nanoparticle materials have different plasmonic resonance frequencies and yield different light scattering spectra. A low surface coverage by metal nanoparticles yields a weak shadowing effect, but also light scattering effect, and *vice versa*.

**Figure 2:**
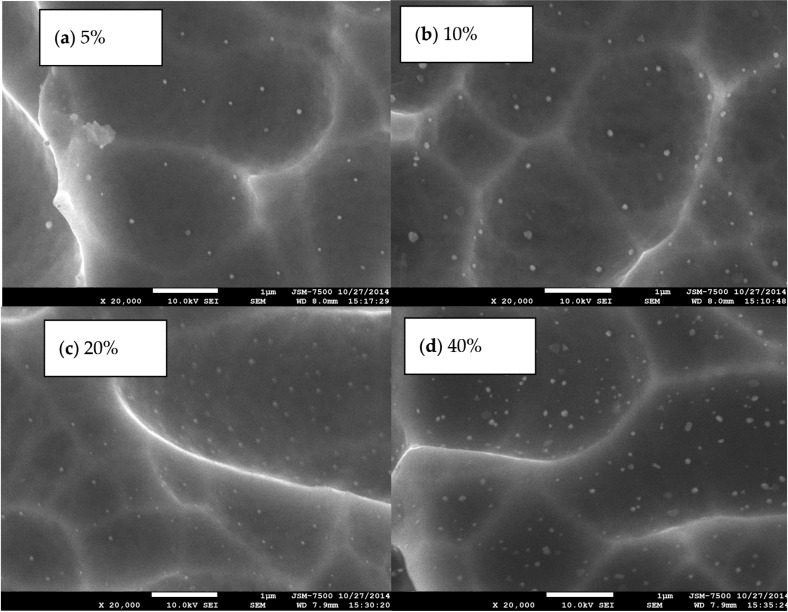
The surface field-emission scanning electron microscope (FESEM) images of Au nanoparticles at a concentration of (**a**) 5%; (**b**) 10%; (**c**) 20% and (**d**) 40% on mc-Si solar cells.

**Figure 3:**
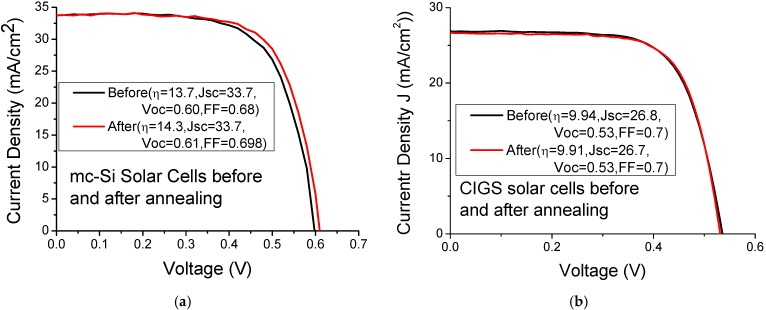
Current-voltage curves of (**a**) mc-Si and (**b**) copper-indium-gallium-diselenide (CIGS) solar cells before and after thermal annealing under AM1.5 illumination.

Figure 2 presents the field-emission scanning electron microscope (FESEM) images of Au nanoparticles on multicrystalline silicon (mc-Si) solar cells. Clearly, the nanoparticles vary in size from 100 nm to several hundred nanometers. The original size of Au-nanoparticles is approximately 100 nm. The spinning and annealing of Au-nanoparticles causes some of them to agglomerate as a large nanoparticle size. The size of agglomerated Au particles is approximately 100~250 nm, verifying that the broadening shape of the resonance wavelength is caused by random distribution and the non-uniform of the sizes of Au nanoparticle. Clearly, the particle density increases with the Au nanoparticle concentration. Figure 3 plots the current-voltage curves of mc-Si and CIGS solar cells before and after thermal annealing under AM1.5 illumination. The average improved efficiency of five mc-Si solar cells annealed by thermal treatment is 2.6%, as shown in Table 1, owing to slightly increasing the V_oc_ and fill factor of mc-Si solar cells. Our experimental results indicated that cutting the large solar cells into small cells of area reduced their efficiency. The reduction in efficiency is attributable to damage that is caused by dicing saw, which can be partially repaired by thermal annealing. The mc-Si solar cells annealed by thermal treatment slightly increased V_oc_ because the damage that was caused by dicing saw was repaired by thermal annealing. Additionally, thermal annealing reduced the series resistance of solar cells, possibly by reducing the contact and interconnect resistances of silver paste, slightly increasing the fill factor of cells. No apparent photocurrent variation of mc-Si solar cells before and after thermal annealing was observed. The efficiency and photocurrent of CIGS solar cells annealed by thermal treatment do not cause apparent change, as shown in Figure 3.

**Figure 4:**
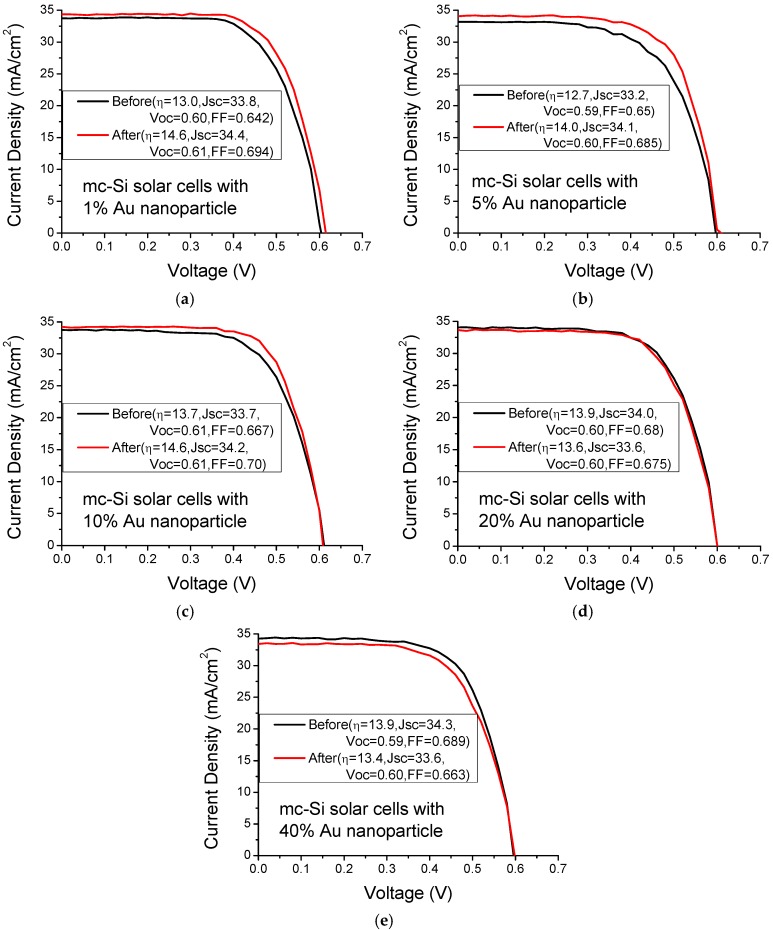
Current-voltage curves of mc-Si solar cells with various Au nanoparticle concentrations of (**a**) 1%; (**b**) 5%; (**c**) 10%; (**d**) 20% and (**e**) 40% under AM1.5 illumination.

Figure 4 plots the current-voltage curves of mc-Si solar cells with various Au nanoparticle concentrations under AM1.5 illumination. The solar cells with Au nanoparticle concentrations of 1%, 5% and 10% exhibit better solar performance than those without metal nanoparticles. However, the solar cells with an Au nanoparticle concentration of 20% and 40% exhibit worse solar performance than those without metal nanoparticles because of the competing effect of metal shadowing and light scattering. Low surface coverage by metal nanoparticles yield a weak shadowing effect, but also reduce weak light scattering. Table 1 presents the average photocurrents and efficiency enhancements of five mc-Si solar cells upon the incorporation of various concentrations of Au and Ag nanoparticles. An Au nanoparticle concentration of 1% can improve the enhancement of solar efficiency in mc-Si solar cells by 8.2%. However, thermal annealing can improve the enhancement of solar efficiency in mc-Si solar cells by 2.6%. Therefore, to exclude thermal annealing effect, the enhancement of solar efficiency in mc-Si solar cells with an Au nanoparticle concentration of 1% was 5.6%, which was improved by the incorporation of Au nanoparticles alone. Nanoparticle incorporation reduced the series resistance of solar cells, possibly by reducing the contact and interconnect resistances of silver paste owing to thermal annealing, slightly increasing the fill factor of cells. The photocurrent increases with Au nanoparticle concentration up to 10%, beyond which it declines. Solar cells with an Au nanoparticle concentration of 10% exhibited the highest enhancement of photocurrent by 1.2%.

**Table 1:** The average photocurrents and efficiency enhancements of five mc-Si solar cells upon the incorporation of various concentrations of Au and Ag nanoparticles.

Enhancement Factor	Nanoparticle Concentrations	Annealed Samples	1%	5%	10%	20%	40%
Au	Jsc ((J_sc(Au)_ − J_sc_/J_sc_)	< 0.1%	1%	1.1%	1.2%	−1.1%	−1.6%
	Eff((E_ff(Au)_ − E_ff_)/E_ff_)	2.6%	8.2%	4.6%	5.9%	−0.2%	−4.9%
Excluded thermal annealing effect, Eff			5.6%	2%	3.3%	−2.8%	−7.5%
Ag	Jsc ((J_sc(Ag)_ − J_sc_)/J_sc_)	< 0.1%	0.6%	0.2%	−0.1%	−2.4%	−6.3%
	Eff((E_ff(Ag)_ − E_ff_)/E_ff_)	2.6%	4.8%	7.4%	2.6%	0.1%	−5.8%
Excluded thermal annealing effect, Eff			2.2%	4.8%	0%	−2.5%	−8.4%

**Figure 5:**
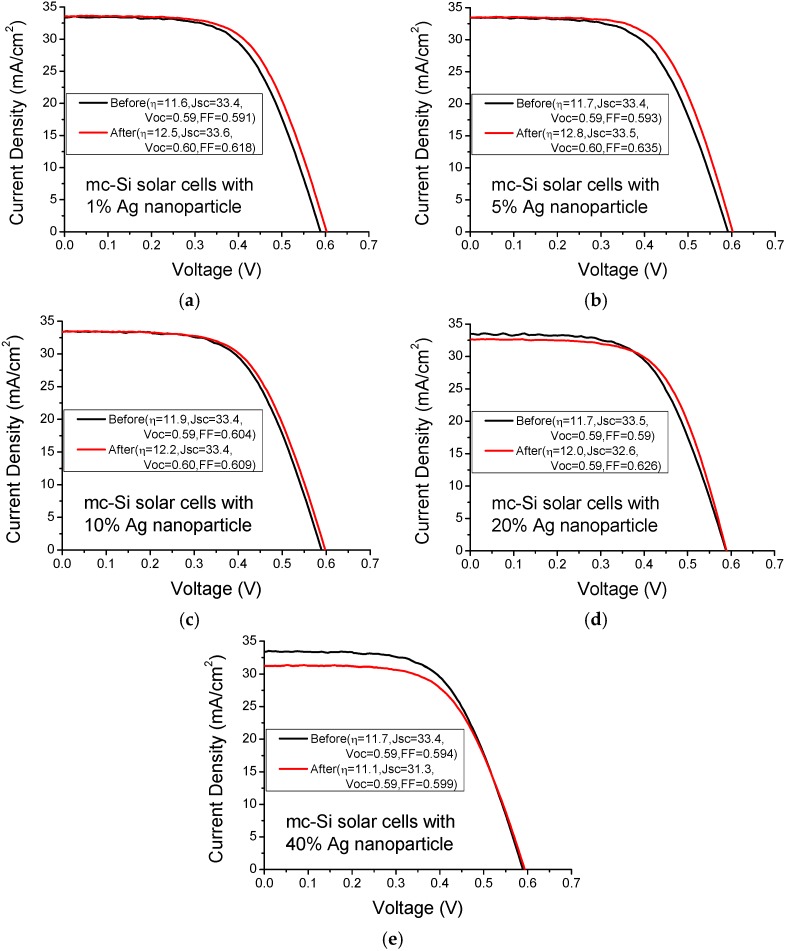
Current-voltage curves of mc-Si solar cells with various Ag nanoparticle concentrations of (**a**) 1%; (**b**) 5%; (**c**) 10%; (**d**) 20% and (**e**) 40% under AM1.5 illumination.

Figure 5 plots the current-voltage curves of mc-Si solar cells with various Ag nanoparticle concentrations under AM1.5 illumination. Like those with Au nanoparticles, solar cells with Ag nanoparticles at concentrations of 1%, 5% and 10% exhibit better solar performance than those without incorporated nanoparticles. Solar cells with an Ag nanoparticle concentration of 20% and 40% exhibit poor solar performance, also owing to the competition between metal nanoparticle shadowing and light scattering. As shown in Table 1, an Ag nanoparticle concentration of 5% improves the efficiency enhancement of mc-Si solar cells by 7.4%.By excluding thermal annealing effect, the enhancement of solar efficiency in mc-Si solar cells with an Ag nanoparticle concentration of 5% was improved by 4.8%. The photocurrents of solar cells with Ag nanoparticle concentrations of 1% and 5% are higher than that without Ag nanoparticles. The photocurrent of solar cells with Ag nanoparticles at a concentration of 1% exhibits the greatest enhancement of 0.6%. Interestingly, mc-Si solar cells with incorporated Au nanoparticles exhibit better solar performance than that with incorporated Ag nanoparticles. This finding is consistent with the observed transmittance of Au and Ag nanoparticles in Figure 1. Like light scattering, the surface plasmonic guiding mode and localized surface plasmonic resonance significantly affect the absorption.

**Figure 6:**
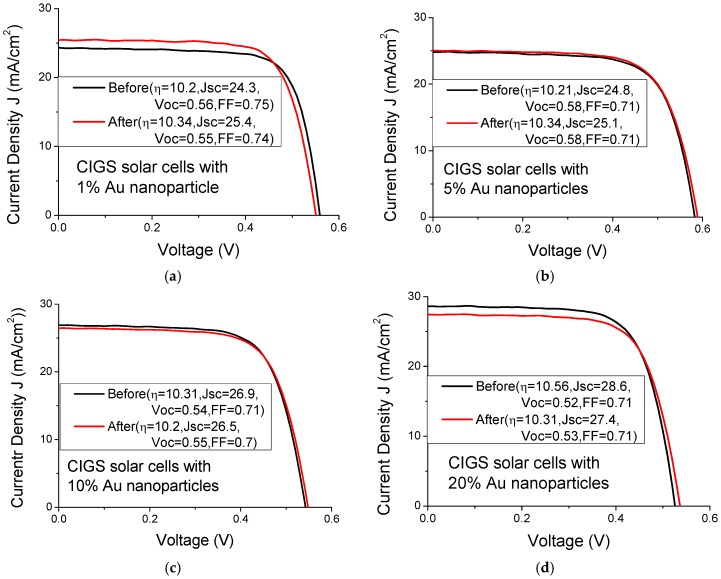
Current-voltage curves of CIGS solar cells with various Au nanoparticle concentrations of (**a**) 1%; (**b**) 5%; (**c**) 10% and (**d**) 20%.

**Figure 7:**
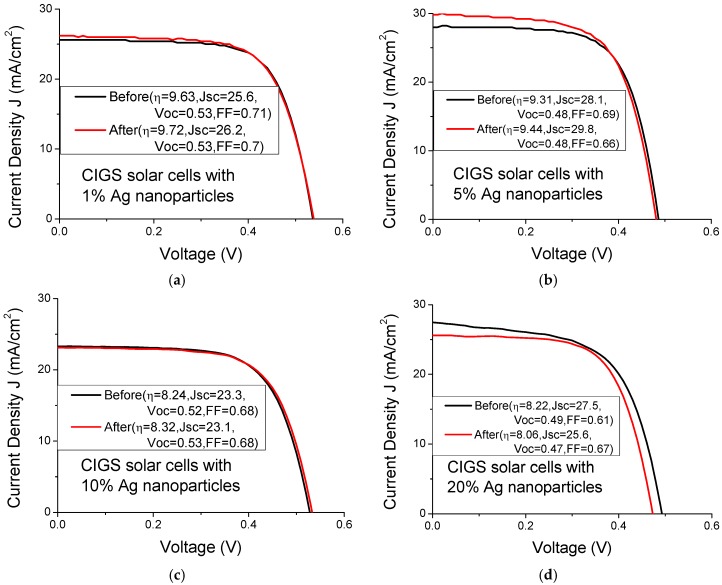
Current-voltage curves of CIGS solar cells with various Ag nanoparticle concentrations of (**a**) 1%; (**b**) 5%; (**c**) 10% and (**d**) 20%.

Figure 6 and Figure 7 plot the current-voltage curve of CIGS solar cells with various Au and Ag nanoparticle concentrations, respectively. Table 2 presents the mean photocurrent and efficiency enhancement of three CIGS solar cells upon the incorporation of various concentrations of Au and Ag nanoparticles. The highest photocurrent enhancement (5.9%) and efficiency enhancement (1.2%) in the CIGS solar cells are achieved at an Au nanoparticle concentration of 1%. However, the highest photocurrent enhancement (2.3%) and efficiency enhancement (1.4%) that are obtained using Ag nanoparticles are achieved at concentrations of 1% and 5%, respectively. The solar cells with a low nanoparticle concentration exhibited better solar performance than those with a high the nanoparticle concentration, indicating that the shadowing effect at high nanoparticle concentration is strong. The enhancement in the solar performance of CIGS solar cells does not differ significantly between the incorporation of Au nanoparticles and that of Ag nanoparticles.

**Table 2:** The mean photocurrent and efficiency enhancement of three CIGS solar cells upon the incorporation of various concentrations of Au and Ag nanoparticles.

Enhancement Factor	Nanoparticle Concentrations	Annealed Samples	1%	5%	10%	20%
Au	Jsc ((J_sc(Au)_ − J_sc_/J_sc_)	< 0.1%	5.9%	3.7%	−2.3%	−3.1%
	Eff((E_ff(Au)_ − E_ff_)/E_ff_)	< 0.1%	1.2%	1.1%	−0.3%	−1%
Excluded thermal annealing effect, Eff			~1.2%	~1.1%	~−0.3%	~−1%
Ag	Jsc ((J_sc(Ag)_ − J_sc_)/J_sc_)	< 0.1%	2.3%	1.9%	−3.3%	−4.3%
	Eff((E_ff(Ag)_ − E_ff_)/E_ff_)	< 0.1%	0.5%	1.4%	−0.1%	−2.1%
Excluded thermal annealing effect, Eff			~0.5%	~1.4%	~−0.1%	~−2.1%

Photocurrent enhancement in mc-Si solar cells is weaker than that in CIGS solar cells. The main geometrical difference between mc-Si and CIGS solar cells is that nanoparticles are located on a texture surface in silicon solar cells and on smooth surface in CIGS solar cells. Metal nanoparticles are well known to increase the efficiency of solar cells by reducing reflection and increasing light trapping. However, metal nanoparticles on flat surface cause high reflectivity losses due to the backscattering of the nanoparticles itself. Tan *et al.* reported that the backscattering effect of nanoparticles can be suppressed by depositing them onto glass sub-wavelength structures, and that doing so greatly reducing reflections from those structures [22]. This geometric texture effect enhances the photocurrent in mc-Si solar cells. Additionally, light is well known to scatter preferentially into a dielectric with higher permittivity when metal nanoparticles are close to an interface between two dielectrics. The relative permittivities of silicon, silicon nitride, CIGS and zinc oxide are 11.9, 8, 13.6 and 9, respectively [23,24]. Therefore, more light is scattered into CIGS solar cells than in mc-Si solar cells when nanoparticles are incorporated on the surface of mc-Si solar cells. The absorption coefficient of CIGS materials is much higher than that of silicon materials. Therefore, the photocurrent enhancement in CIGS solar cells is larger than that of mc-Si solar cells. The top surface of mc-Si and CIGS solar cells is at least 500 nm away from the p/n junction interface in these two solar cells. We believed that the localized surface plasmonic resonance effect that enhances absorption arises from the fact that the near-field intensities at the p/n junction are small and similar in these two solar cells. The large photocurrent enhancement in CIGS solar cells may follow from the fact that exhibit greater scattering of light and a higher absorption coefficient than mc-Si solar cells.

## 3. Experimental Section

Mc-Si solar cells with a size of 16 cm × 16 cm were obtained from Solartech Energy Corporation of Taiwan. Each large size solar cell was cut into small cells with a size of 2 cm × 2 cm. The thickness of silicon nitride antireflection coating layer was approximately 70 nm and the depth of the region that was highly n^+^ doping by phosphorus was approximately 500 nm. For fabricating CIGS solar cells, a piece of soda-lime glass with an area of 30 cm × 30 cm was cut into small pieces with an area of 4 cm × 4 cm. These glass substrates were ultrasonically cleaned in a detergent bath, and then in an acetone, isopropanol and deionized water, each for 15 min. After cleaning, a 1 μm-thick Mo layer was deposited on the soda-lime glass by DC magnetron sputtering. The CIGS absorber layer was prepared by three-step co-evaporation. In the first step, the selenium (Se) was heated to 270 °C to evaporate Se as a gas. The substrate was heated at 530 °C, and gallium (Ga) and Indium (In) were co-evaporated by two heaters with the working current of 15.2 and 13.6 A, respectively. The Ga and In co-evaporations were terminated after 17 min. In the second step, Se gas was supplied continuously and copper (Cu) was evaporated at the heater with the working current of 18.6 A, until a Cu-rich film was detected by end-point detection [25]. In the third step, gaseous Se was supplied continuously, and Ga and In were co-evaporated for 6 min to obtain Cu-poor CIGS thin films. The Ga/(Ga + In) composition ratios of the CIGS films were approximately 0.31 and the thickness of the CIGS films was approximately 1.7 μm. A CdS buffer layer with a thickness of 50–60 nm was prepared by chemical bath deposition (CBD). The intrinsic zinc oxide (i-ZnO, 80 nm)/Al-doped zinc oxide (AZO, 350 nm) layers were sequentially deposited by RF magnetron sputtering. Finally, a grid of Ni (0.05 μm)/Al (2 μm) was deposited by e-beam evaporation. Sixteen CIGS solar cells with a soda-lime glass/Mo/CIGS/CdS/i-ZnO/AZO/(Ni/Al grid) structure were fabricated on a size of 4 cm × 4 cm. The active area of CIGS solar cells is 0.358 cm^2^. Before spin coating and thermal annealing, current-voltage (I–V) measurement of 55 mc-Si and 27 CIGS solar cells (5 mc-Si and 3 CIGS solar cells for thermal annealing, 25 mc-Si and 12 CIGS solar cells for Au spin coating (5 mc-Si and 3 CIGS solar cells for each nanoparticle concentration), 25 mc-Si and 12 CIGS solar cells for Ag spin coating (5 mc-Si and 3 CIGS solar cells for each nanoparticle concentration)) was carried out by a Yamashita DensoYSS-50Aunder AM1.5 illumination.

Concentrated solutions of Au and Ag nanoparticles with a size of approximately 100 nm were purchased from Xuan-Bao-Hao Chemical Corporation of Taiwan. The Au/Ag nanoparticle solution was diluted by adding methylbenzene to yield Au/Ag nanoparticle concentrations of 1%, 5%, 10%, 20% and 40%. The diluted solution was spun on the surfaces of five mc-Si and three CIGS solar cells for each nanoparticle concentration. Before the solutions were spun, the solar cells had been ultrasonically cleaned in a detergent bath, then in an acetone, isopropanol and deionized water, each for 5 min. The initial rate of spinning of the nanoparticle solutions was 1000 rpm, which was applied for 10 s, and the final spinning speed was 3000 rpm, which was applied for 30 s. Thereafter, the solar cells were dried at 100 °C on hot plate for 5 min and then annealed in a furnace tube at 280 °C for one hour in nitrogen gas to remove the solvents. In order to understanding thermal annealing effect, five mc-Si and three CIGS solar cells were annealed at 280 °C for one hour in nitrogen gas for comparison. After spin coating and thermal annealing, I–V measurement of 55 mc-Si and 27 CIGS solar cells was performed again. The enhancement of efficiency and photocurrent in these solar cells was calculated by I–V measurement before and after spin-coating (or thermal annealing) solar cells. The average photocurrents and efficiency enhancements of five mc-Si and three CIGS solar cells upon the incorporation of various concentrations of Au and Ag nanoparticles were presented in Table 1 and Table 2, respectively. The photocurrents and efficiency enhancements of spin-coating solar cells included the incorporating nanoparticle and thermal annealing effects. To exclude thermal annealing effect, the photocurrents and efficiency enhancements of spin-coating solar cells minus the enhancements of thermal annealing solar cells leave the enhancements, which are improved by the incorporating nanoparticle alone. Metal nanoparticle distribution was studied by field-emission scanning electron microscopy (FESEM) with JEOL JSM-7005F. The UV-vis transmittance spectra were recorded with a JASCO V-670 Spectrophotometer.

## 4. Conclusions

A simple spin-coating process for incorporating Au and Ag nanoparticles on mc-Si and CIGS solar cells was studied. Incorporating Au/Ag nanoparticles at suitable concentrations on mc-Si and CIGS solar cells improved their efficiency enhancement by 5.6%/4.8% and 1.2%/1.4%, respectively. Although the energy bandgap of silicon is close to those of the CIGS materials, the enhancement of the efficiency of silicon cells upon the incorporation of Au/Ag nanoparticles differs greatly from that of CIGS solar cells, perhaps because of their different light scattering behaviors and material absorption coefficients.
